# Barbara Epstein, AHIP, FMLA, Medical Library Association President, 2017–2018

**DOI:** 10.5195/jmla.2017.304

**Published:** 2017-10-01

**Authors:** Kelly Gonzalez

**Figure:**
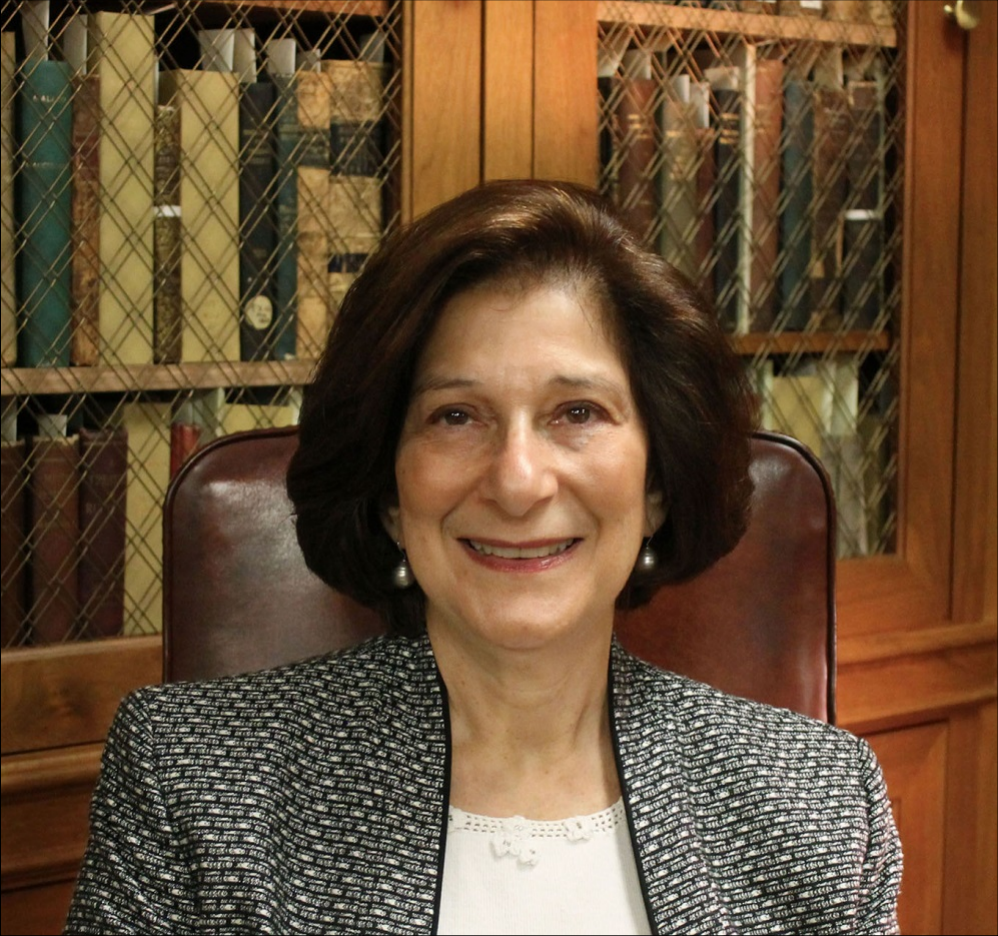


Meeting Barbara Epstein, AHIP, FMLA, for the first time at the 2010 Medical Library Association (MLA) annual meeting in Washington, DC, was very memorable. That was the meeting that cardboard cutouts of President Barack Obama and First Lady Michelle Obama greeted everyone at the top of the Hilton Washington escalator. It was also the meeting that Earvin “Magic” Johnson Jr. made an appearance at the Hilton for a separate, non-MLA-related, event. I was a new National Library of Medicine (NLM)/Association of Academic Health Sciences Libraries (AAHSL) Fellow, and Barbara was selected as my mentor. I had no idea who she was; I needed to do my research.

My library director at that time, Laurie L. Thompson, AHIP, FMLA, took great care to introduce me as a Fellow to respected MLA members like Jett McCann, AHIP, senior associate dean for knowledge management and director of the Dahlgren Memorial Library at Georgetown University Medical Center, who participated in the Fellows’ capstone event, and Carolyn E. Lipscomb, AHIP, FMLA, former program manager of the AAHSL Future Leadership Committee, along with many others. Each introduction of me as a Fellow included the mention of Barbara Epstein as my upcoming mentor and the reactions were all positive: “Very good!” “Wonderful!” “Great!” “You are in good hands” and “Take advantage of this opportunity.” It was Laurie Thompson who arranged for us to have dinner with Barbara and two of her staff members, Melissa Ratajeski, AHIP, and Jill Foust.

The topic of MLA oral histories was a common thread for Barbara and me. It was 2010 when I became a member of the MLA Oral History Committee that I would later chair. Barbara spoke eloquently of the importance of oral histories in her 2015 Janet Doe Lecture, “In Their Own Words: Oral Histories of Medical Library Association Past Presidents.”

“Natural and progressive leadership” were words that described Barbara when she was honored in 2016 as an MLA Fellow. As chair of the Scholarly Communications Committee in 2012/13, she advocated to members of Congress on behalf of the MLA membership. She had been a member of the MLA/AAHSL Joint Legislative Task Force from 2008–2016. Ruth Riley, AHIP, AAHSL president in 2015/16, served with Barbara on the AAHSL Board during 2014–2015 and grew to greatly appreciate her ability to approach issues in a systematic, strategic way. Ruth Riley was impressed with Barbara’s keen understanding of how issues facing the association fit into the context of the complex environment and politics of academic health centers. Ruth Riley said that Barbara is a master at taking an issue and turning it into a strategic action plan that serves our profession and, ultimately, our stakeholders well.

During her year as MLA president-elect in 2016/17, Barbara served on the Board of Directors and Executive Committee. Fellow Board Member Lisa K. Traditi, AHIP, was impressed with her ability to hear a variety of opinions and distill from them what really matters. During one of her first board meetings, several members were voicing strong opinions about a pending decision. Barbara’s ability to ask probing questions and quickly get to the root of the various concerns was appreciated. She listens carefully and communicates clearly, talents which make her a strong leader. As a Fellow, I spent two separate weeks in Pittsburgh being mentored by Barbara, observing her management and leadership style and watching as her assistant directors worked cooperatively with her to develop new problem-solving approaches. I learned so much.

Barbara chaired the MLA Leadership and Management Section (LMS) in 2007/08 and was treasurer in 1999–2000. She participated in planning the transition of the former Medical School Libraries Section to the more dynamic LMS; chaired the Continuing Education Committee in 1995/96; chaired the Mental Health Librarians Section in 1978/79 and was treasurer in 1986–1988; and chaired the Pittsburgh Chapter from 1986–1988, successfully advocating for its merger with the Mid-Atlantic Chapter. MLA bestowed the honor of Fellow to Barbara based on her accomplishments and contributions to the health sciences information profession. As mentioned previously, she was awarded the 2015 Janet Doe Lectureship, through which she communicated her broad perspective on medical librarianship. In 1981, the Ida and George Eliot Prize was awarded to her and her coauthors for a seminal work, “JCAH Accreditation and the Hospital Library: A Guide for Librarians.”

Barbara is the director of the Health Sciences Library System (HSLS) of the University of Pittsburgh, as well as the director of the Middle Atlantic Region (MAR) of the National Network of Libraries of Medicine (NNLM). In 2010, Barbara and her team successfully applied for the contract to serve as the Regional Medical Library for the NNLM MAR. She was the associate director at HSLS from 1995–2003 and beginning in 1998, a member of the faculty for the Medical Informatics Training Program in the Department of Biomedical Informatics at the University of Pittsburgh School of Medicine. During my time at HSLS, I met with many people outside of the library, and it was clear that Barbara knew many people at University of Pittsburgh Medical Center and University of Pittsburgh. These relationships and the community that she had built through the years developed trust in her and, therefore, inclusion of her in strategy and implementation or change management.

Barbara was also the director, 1985–1995, associate director, 1984–1985, and reference librarian, 1978–1984, at the Western Psychiatric Institute and Clinic (WPIC) Library, University of Pittsburgh Medical Center (UPMC). Early in her career, she gained experience in many different aspects of librarianship, including indexing and cataloging items in the WPIC collection, providing reference assistance, developing and teaching library students, and fulfilling library management responsibilities. During my time in Pittsburgh as a Fellow, she told me of the closure of the WPIC Library, of how she communicated with their users and relocated the resources. I remember her pointing out the building that had housed WPIC and taking the time to flesh out the details, to help me understand the complexity of issues surrounding not only the library, but WPIC itself.

Barbara’s undergraduate degree is in French and political science from the University of Pittsburgh. She received her master of science in library science (MSLS) degree from Case Western Reserve University. The only thing that the University of Pennsylvania and University of Pittsburgh have in common is that they are in the same state. There is no mistaking that Barbara is a University of Pittsburgh fan, along with MLA colleague Janice E. Kelly, FMLA. Barbara and her husband, Arnold, have two adult daughters, Margo and Lauren. These two professional women, whom I have never met but I feel like I know, practice medicine and law. Along with Pitt athletics, the grandchildren also take a bit of priority.

In Barbara’s own words, “Change has been an integral part of my career. The key to leading organizational change is the recognition that it must be a team effort.” As a director of two major libraries at the University of Pittsburgh, WPIC and HSLS, she helped stretch boundaries and develop new services. Through her contributions to MLA so far, she notes that “leading organizational change can be challenging, exciting, and sometimes even exhilarating. However difficult and risky change may seem, it is an even greater risk to stay in place.”

